# The Modulation by the Locus Coeruleus of Recent and Remote Memory Retrieval is Activity‐Dependent

**DOI:** 10.1002/hipo.70004

**Published:** 2025-02-20

**Authors:** Natalia Babushkina, Denise Manahan‐Vaughan

**Affiliations:** ^1^ Medical Faculty, Department of Neurophysiology Ruhr University Bochum Bochum Germany; ^2^ International Graduate School of Neuroscience Ruhr University Bochum Bochum Germany

**Keywords:** dopamine, hippocampus, locus coeruleus, memory retrieval, noradrenaline

## Abstract

The hippocampus plays a crucial role in acquiring, storing, and retrieving associative experience. Whereas neuromodulatory control of the hippocampus by the locus coeruleus (LC) enhances memory acquisition and consolidation, less is known about its influence on memory retrieval. The LC fires at tonic (0.5–8 Hz) and phasic frequencies (10–25 Hz), relative to arousal and affective states. Here, we explored to what extent LC stimulation at different frequencies (2–100 Hz) and respective stimulation patterns, before retrieval of recently acquired or remote spatial memory, alter working memory (WM) or reference memory (RM) in male rats. Here, animals learned a spatial memory task in an eight‐arm radial maze over a period of 15 days. LC stimulation before recent memory testing did not affect WM. However, LC stimulation at 20 or 100 Hz, but not 5–10 Hz, impaired retrieval of recently consolidated RM. These frequency‐dependent impairments were abolished by intracerebral β‐adrenergic receptor (β‐AR), but not D1/D5 receptor, antagonism. When memory retrieval was assessed 4 weeks after initial consolidation (Day 34), RM was significantly impaired compared to the final day of recent memory testing (on Day 6). RM was not altered by LC stimulation before remote memory retrieval. However, LC stimulation at 2–100 Hz improved WM. Taken together, these data suggest that frequency‐dependent NA release from the LC disrupts retrieval of recently acquired RM via activation of β‐AR. Strikingly, increasing LC activity *in general* improves WM of a remotely acquired spatial learning task, assessed 4 weeks after the recent memory testing, suggesting that the increased effort of sustaining WM of a task learned in the past requires higher LC engagement.

## 1 Introduction

The ability to select, store, retrieve, and update information supports prospective behavior and adaptation to a dynamically changing environment. The hippocampus is a primary brain structure for experience‐dependent information encoding and updating (Manahan‐Vaughan 2017) that is supported in information selection and retrieval by subcortical and cortical structures (Stacho and Manahan‐Vaughan 2022). The locus coeruleus (LC) supports both arousal‐related and cognitive information processing by the brain (Poe et al. 2020) and potently regulates experience‐dependent information processing by the hippocampus (Hagena et al. 2016; Babushkina and Manahan‐Vaughan 2022). In addition to its role as the sole source of noradrenaline (NA) for the brain, it releases other neuromodulators such as dopamine (Smith and Greene 2012; Lemon and Manahan‐Vaughan 2012; Kempadoo et al. 2016; Takeuchi et al. 2016) in an activity‐dependent manner (Babushkina and Manahan‐Vaughan 2022). Both β‐adrenergic receptors (β‐AR) and dopaminergic D1/D5 receptors (D1/D5R) are particularly involved in the acquisition and/or consolidation of hippocampus‐dependent memory and also regulate the magnitude and persistence of hippocampal synaptic plasticity (Lemon and Manahan‐Vaughan 2006; Lemon et al. 2009; Lemon and Manahan‐Vaughan 2012; Hansen and Manahan‐Vaughan 2015; Babushkina and Manahan‐Vaughan 2022).

The LC exhibits phasic (transient 10–20 Hz bursts) and tonic (0.1–7 Hz) neuronal activity (Aston‐Jones and Cohen 2005; Berridge and Waterhouse 2003; Devilbiss and Waterhouse 2011) that correspond to behavioral states. For example, phasic activity occurs in response to exposure to novel experiences or to changes in incoming information (Aston‐Jones and Bloom 1981b; Sara et al. 1994), while tonic firing frequencies occur during waking states (Aston‐Jones and Bloom 1981a), prolonged attention (Aston‐Jones et al. 1999), and task disengagement during problem solving (Aston‐Jones and Cohen 2005). Interestingly, phasic LC firing is closely related to attentional and task performance, whereas elevated tonic activity may correspond to poor task performance (Aston‐Jones et al. 1999).

Most studies that have addressed the role of the LC in memory have focused on acquisition (Lemon et al. 2009; Kempadoo et al. 2016; Wagatsuma et al. 2018; Tsetsenis et al. 2022), or consolidation processes (LaLumiere et al. 2003; Novitskaya et al. 2016; Takeuchi et al. 2016).

Fewer studies have examined the role of LC in memory retrieval. Studies in rodents and humans have demonstrated that noradrenergic neuromodulation is necessary for this process (Sterpenich et al. 2006; Murchison et al. 2004; Korz and Frey 2007; Sara and Devauges 1988; Devauges and Sara 1991). In particular, it has been reported that NA receptor activation is required for the retrieval of spatial memory and contextual fear memory (Murchison et al. 2004; Korz and Frey 2007; Quyang and Thomas 2005), whereas others have reported that the activation of β‐AR impairs memory retrieval (Qi et al. 2008; Grella et al. 2021). Given that the LC is the primary source of NA for the brain, it is probable that the abovementioned processes, albeit unresolved, may be mediated by the LC.

The involvement of the LC in memory consolidation and retrieval is very likely to be dependent on frequency and patterns of LC discharge: whereas LC stimulation, in the mid‐phasic range of 20 Hz during sleep, has no effect on the consolidation of spatial memory, high‐phasic stimulation of the LC at 100 Hz interferes with sleep consolidation of spatial reference memory (RM) (Novitskaya et al. 2016). By contrast, optogenetic stimulation of tyrosine hydroxylase‐expressing neurons of the LC at 25 Hz promotes D1/D5R‐dependent choice memory (Tse et al. 2023) and electrophysiological stimulation at 100 Hz promotes β‐AR‐dependent episodic‐like memory in rats (Lemon et al. 2009). In addition, although stimulation of the LC at 2, 5, 10, or 100 Hz, in conjunction with test‐pulse stimulation of hippocampal afferents, results in *N*‐methyl‐d‐aspartate receptor (NMDAR)‐dependent hippocampal LTD (Lemon et al. 2009; Babushkina and Manahan‐Vaughan 2022), 25 Hz stimulation of the LC during urethane anesthesia of rats results in slow‐onset potentiation (Tse et al. 2023).

In the present study, we aimed to elucidate to what extent the LC is involved in the retrieval of both well‐consolidated recent and remote spatial memory. To assess the frequency‐dependency of this process, we examined memory retrieval while stimulating the LC electrophysiologically at 2, 5, 10, 20, or 100 Hz to emulate its activity in the range of its tonic and phasic firing rates. We also assessed to what extent the involvement of the LC in these memory processes depends on β‐AR or D1/5R. Animals were initially tested after recent training in an 8‐arm radial maze and then tested once more 4 weeks after successful learning of the task. We observed that firing frequencies related to phasic LC activity interfered with the retrieval of recently acquired RM of the spatial learning task and that effects were β‐AR but not D1/5R‐dependent. One day later, RM had returned to post‐acquisition levels, suggesting that LC activation served to distract from the task without affecting consolidation. By contrast, phasic stimulation of the LC *promoted* the retrieval of remote RM 4 weeks after task acquisition. Furthermore, whereas stimulation of the LC did not affect working memory (WM) during the completion of the recently acquired spatial learning task, stimulation of the LC in the range of 2–10 Hz improved WM during participation in the remotely acquired task. These findings suggest that phasic LC activity immediately before the retrieval of recent memory may divert attention from retrieval cues, but nonetheless leave consolidated memory intact. By contrast, phasic LC activity immediately before the retrieval of remote memory may conversely promote focused attention to the task cues during increased cognitive effort.

## 2 Materials and Methods

All experimental procedures were approved in advance by the ethics committee of the federal state of NorthRhine Westphalia (NRW) (Landesamt für Naturschutz, Umweltschultz und Verbraucherschutz, NRW, Bezirksamt Arnsberg) and carried out according to the guidelines of the European Communities Council Directive of September 22, 2010 (2010/63/EU) for the care of laboratory animals.

### 2.1 Animals

Male Wistar rats (Charles River, Germany, 9–10 weeks old) were used for this study. The animals were housed in a rodent vivarium (Scantainer, Scanbur Technology A/S, Denmark) with a 12 h light/12 h dark cycle (lights on from 7 a.m. to 7 p.m.) at a temperature of 22°C ± 2°C and humidity of 55% ± 5%, with *ad libitum* access to water. At least 1 week before the beginning of the experiment, animals were food‐restricted to decrease their weight by 10%–15% of their initial pre‐habituation weight. This weight was then monitored daily and maintained for the duration of the experiments. During this time, the animals underwent daily health checks and handling by the experimenter and were habituated to the plugging and unplugging of the connector for electrophysiology.

### 2.2 Surgery

Under sodium pentobarbital anesthesia (52 mg/kg, intraperitoneally), the animals were chronically implanted with a stimulating electrode in the LC and a guide cannula, as described previously (Babushkina and Manahan‐Vaughan 2022). For this, a bipolar stimulating electrode was implanted unilaterally into the LC of the right hemisphere (coordinates: 3.3 ± 0.1 mm posterior to lambda, 1.2–1.3 mm lateral to the midline and at a 15° angle, relative to the skull's plane). To note: the 15° angle means that the final location of the electrode tips in the LC does not align with the plate numbers in the rat brain atlas used to determine the initial insertion coordinates of the electrodes, as described above (Paxinos and Watson 2014). The final dorsoventral position of the electrode was determined by the detection of twitching of vibrissae following LC stimulation at 100–400 μA (Eschenko 2018). To enable pharmacological treatment, the guide cannula was implanted ipsilaterally into the lateral ventricle (coordinates: 0.5 mm posterior to bregma, 1.6 mm lateral to the midline). The electrode and the cannula were permanently attached to the skull with dental acrylic (Paladur, Heraeus Kulzer GmbH, Hanau, Germany). Animals were treated subcutaneously with the analgesic Meloxicam (Metacam, 0.2 mg/kg, Boehringer Ingelheim Vetmedica GmbH, Ingelheim am Rhein, Germany) before and after surgery. Postmortem verifications of electrode and cannula localizations were conducted.

### 2.3 Compounds

The β‐AR antagonist, propranolol (Tocris, Wiesbaden‐Nordenstadt, Germany), was dissolved in NaCl (0.9%) at a dose of 2 μg/μL (total amount administered: 10 μg). The D1/D5R antagonist, SCH23390 (Tocris, Wiesbaden‐Nordenstadt, Germany), was dissolved in 0.9% NaCl to obtain a dose of 5.94 μg/μL (total amount administered: 29.7 μg). These doses were previously shown to modulate synaptic plasticity at perforant path‐dentate gyrus synapses of freely behaving rats, while having no effect on basal synaptic transmission (Hansen and Manahan‐Vaughan 2015; Wiescholleck and Manahan‐Vaughan 2014). A total volume of 5 μL was administered via the guide cannula by using a fine‐gauge syringe (Hamilton Company, Reno, NV) attached to an inner cannula via polyethylene tubing. All injections were carried out over a 5‐min period, 30 min before commencing the LC stimulation protocol. After injections, the inner cannula was left in place for 1–2 min to ensure that the solution had exited the tubing and inner cannula.

### 2.4 Locus Coeruleus Stimulation

One week before the experiments, the appropriate intensity for LC stimulation was individually chosen for each animal through the assessment of behavior in response to current injection, as described previously (Lemon et al. 2009; Hansen and Manahan‐Vaughan 2015). The selected stimulus strength was in the range of 30–180 μA.

The current intensity used for LC stimulation was determined independently for each rat by selecting the level of LC stimulation that was subthreshold for triggering behavioral responses such as freezing, production of fecal boli, or a head twitch. We also took into consideration the current intensities that were used in the previous studies (Lemon et al. 2009; Sara and Devauges 1988). Since the trigeminal nucleus and the LC are located adjacently to one another and it has been shown that the LC projects to the mesencephalic trigeminal nucleus in rats (Takahashi et al. 2010) a stimulation intensity that is too high results in vibrissae twitching due to current spread to the trigeminal nucleus. This was also monitored carefully and stringently avoided when selecting the stimulation intensity.

The following protocols for LC stimulation were applied to reflect “tonic” stimulation (all with pulse durations of 0.2 ms): 2 Hz for 20 s, 5 Hz for 20 s, 10 Hz for 10 s. For “phasic” stimulation, 20 Hz (comprising 10 trains of six pulses, each of 0.5 ms duration, at 1 s intertrain intervals) and 100 Hz (2 trains of 100 pulses each of 0.2 ms duration) were applied, with each train lasting 1 s and a 20 s intertrain interval. It was shown previously that the latter protocol, when applied electrophysiologically to the LC, induces input‐specific LTD in freely behaving rats if the protocol is applied in conjunction with test‐pulse stimulation of either Schaffer‐collateral‐CA1 synapses or perforant path‐dentate gyrus synapses (Hansen and Manahan‐Vaughan 2015; Lemon et al. 2009; Babushkina and Manahan‐Vaughan 2022).

### 2.5 Behavioral Experiments

**FIGURE 1:**
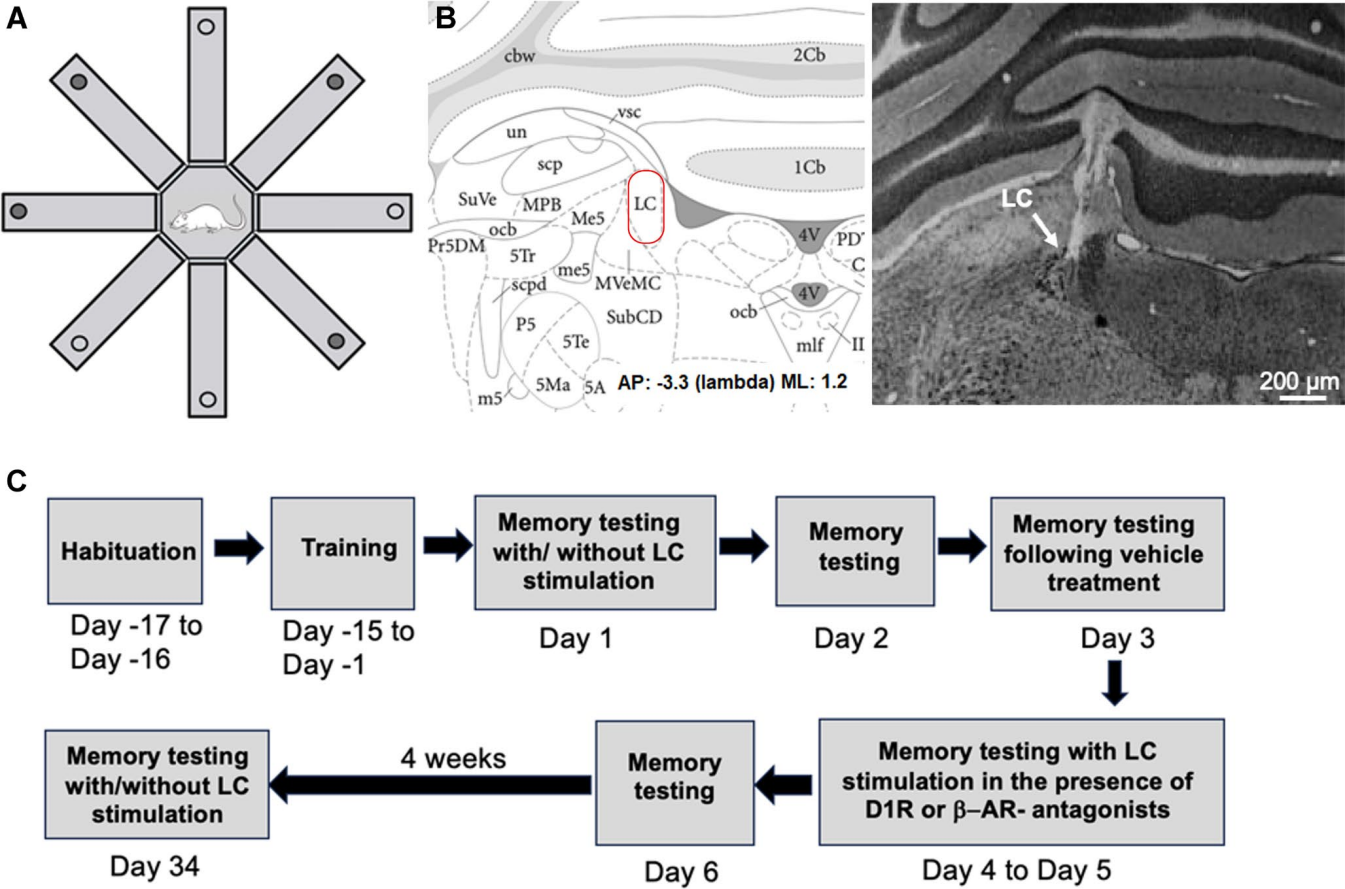
Schema of radial maze, examples of electrode localizations, and experimental protocol. (A) The 8‐arm radial maze was 160 cm in diameter. Arm width was 12 cm. Two odorless pellets were placed in an indentation at the end of the arm. Four arms contained pellets at the beginning of each training or test trial. The arms were selected randomly for each animal and remained consistent during all test trials. (B) Histological examples of electrode localizations in the locus coeruleus (LC). Brain section showing location of LC and Nissl‐stained histological section of the LC. Arrow points to the location of bipolar recording electrodes in the LC (adapted from Paxinos and Watson 2014). (C) Timeline of the experimental protocol. Following 2 days of habituation, during which time multiple pellets were present at the end of each radial maze arm, training began. Rats were trained in an 8 arm radial maze, where four of the eight arms were baited with the reward pellets. Two types of errors were evaluated: working memory (WM) errors, when rats re‐entered the arm from which pellets had already been retrieved within a current trial, and reference memory (RM) errors when animals visited a never‐baited arm. Once animals had reached learning criteria, WM and RM was tested on Day 1 when animals received LC stimulation immediately before recent memory testing in the radial maze. On Day 2, WM and RM were assessed anew, and on Day 3 recent memory testing was repeated 30 min after application of vehicle. On Day 4 and 5 the effect of pharmacological antagonism of D1/D5R, or β‐AR, in the presence of LC stimulation, was tested in different animal cohorts. On Day 6, WM and RM were assessed again, before the retention period was started. After an interval of 4 weeks, during which time the animals did not engage in behavioral manipulations, WM and RM were assessed in the presence, or absence, of LC stimulation. LC stimulation was applied at a range of frequencies: 2, 5, 10, 20, or 100 Hz to different animal cohorts. Five separate animal cohorts were tested, each receiving only one of the LC stimulation protocols. Two control groups were included for the LC tests: animals that had electrodes in the LC, but did not receive stimulation, and animals that had no electrode implantations. All animals had a cannula implanted into the lateral cerebral ventricle through which vehicle, or ligand, treatment was conducted 30 min before recent memory testing began on designated experimental days.

Animals were trained to find and remember the location of odorless food pellets (Dustless Precision Pellets; Bioserv, NJ) (Figure 1). An eight‐arm radial maze task was implemented as previously described (Naie and Manahan‐Vaughan 2004), including the modifications outlined below. The maze was elevated 80 cm from the floor. It consisted of a central octagonal platform (26 cm diameter) with eight arms (67 cm long, 30 cm deep, and 12 cm wide) (Figure 1A). The bottom of the maze was made of dark gray polyvinylchloride, whereas the walls were made of opaque matt polyvinylchloride. The end of each arm included a small circular indentation (1 cm deep, 3 cm diameter), located in the floor of the maze 3 cm from the end of the arm. The hole was deep enough to prevent visibility of the pellets from the central platform of the maze. The end of each arm had no wall. The experimental room was uniformly illuminated to avoid shadowing or light reflections in the maze and had white walls, which were decorated with extra‐maze cues: a black cross (30 × 20 cm) was placed on one of the walls and two black rectangles (50 × 70 cm), 15 cm apart, were positioned on the opposite wall. The position of the extra‐maze cues remained the same across the experiments. To circumvent the animals using intramaze cues to find the baited arms, the maze was thoroughly cleaned and rotated by 45° after each training day.

The experiment began with two consecutive days of habituation to the maze, during which time multiple food pellets were available in all arms of the maze (Figure 1C). Each habituation trial lasted 15 min. On all training and recent, or remote, memory testing days, only four arms were “baited” with two odorless food pellets each (Dustless Precision Pellets, 45 mg, Rodent Purified Diet, Bio‐Serv Inc., Flemington, NJ). For each animal, a different pattern of baited arms was randomly chosen at the start of training, but the pattern remained constant throughout the training and experimental days. Animals participated in four trials per day that were spaced by an interval of 60 min between trials. At the beginning of each training trial, or of recent/remote memory testing, the animal was placed on the central platform of the maze, which was initially restricted by custom‐made “trapping walls.” These walls served to keep the animal in the central platform area during the application of the LC stimulation protocol (or an equivalent delay for controls). The trial commenced when these “trapping walls” were quietly and swiftly removed from the maze to allow the animal a free choice of visits to the arms of the maze. A trial ended after all food pellets were retrieved from the arms, or 15 min had elapsed. After each trial, the maze surface was cleaned to remove olfactory trails generated by conspecific deposits of, for example, saliva, urine, or sebum. The food pellets were not replaced during a trial once they had been retrieved from the arms. Each trial was recorded by a video camera placed above the maze. It typically took 15 days for the animals to achieve the learning criteria.

Rats were considered to have learned the task when all food pellets were collected within 15 min and the RM and WM errors were each < 1 per trial (Roullet and Sara 1998). The animals were then randomly sorted into control and treatment groups, and subsequent statistical analysis confirmed that these cohorts did not differ from one another in terms of their WM and RM learning performances, as well as their velocity and trial latencies (Figure S1). Seven groups were created:

1. LC stimulation at 2 Hz (*n* = 7),
2. LC stimulation at 5 Hz (*n* = 7),
3. LC stimulation at 10 Hz (*n* = 6),
4. LC stimulation at 20 Hz (*n* = 9),
5. LC stimulation at 100 Hz (*n* = 11),
6. A group (Non‐implanted control/“electrode absent”) that did not undergo electrode implantation but followed all procedures (*n* = 8), and
7. A group (Non‐stimulated control/“electrode present”) that underwent electrode implantation but never received LC stimulation (*n* = 6).

On Day 1 of recent memory testing, animals received LC stimulation at either 2, 5, 10, 20, or 100 Hz (Figure 1B) using the protocols described above. LC stimulation protocols were applied immediately before recent memory testing. For that, animals were placed in the central area of the maze, which was restricted by the “trapping” walls while the LC stimulation protocol was applied. Two control cohorts (electrodes present in LC, electrodes absent) did not receive stimulation but followed the same procedures as the stimulated animals.

On Day 2, retention of WM and RM was assessed in all groups. On Day 3, testing of recent memory was repeated 30 min after intracerebral treatment with vehicle for the group stimulated at 20 Hz and the group stimulated at 100 Hz. On Days 4–5, the effect of D1R or β‐AR treatment was assessed in these groups in combination with LC stimulation either at 20 Hz or at 100 Hz. The stimulation protocols were the same as on Day 1, that is, animals that previously received 20 Hz LC stimulation on Day 1 received 20 Hz stimulation on Day 4 and on Day 5. The groups stimulated at 20 Hz and at 100 Hz were counterbalanced for ligand treatment, that is, on Day 4 half of the animals received LC stimulation at 20 Hz in combination with the D1/D5R antagonist and the other half of the animals received LC stimulation at 20 Hz in combination with the β‐AR antagonist. On Day 5, the ligand treatment was reversed. The same counterbalanced procedure was conducted with the group stimulated at 100 Hz. Control animals did not receive stimulation or intracerebral treatment with the ligands or vehicle, but memory performance of these control groups was assessed from Day 1 through Day 6. On Day 6, memory was tested again with all seven groups, as on Day 2, and this performance served as a benchmark for memory assessment after the retention period. After the conclusion of the experiments on Day 6, animals were returned to their home cages for 4 weeks, during which time they were not subjected to any further tests. For the initial 3 weeks of this period, the animals had ad libitum access to food, and in the fourth week, food availability was restricted to achieve a body weight that was 85%–90% of that determined per animal at the end of the third week.

For intracerebral treatment, either 5 μL of vehicle (0.9% NaCl) or an antagonist of either D1R (SCH23390; 5.94 μg/μL) or β‐AR (propranolol; 2 μg/μL) was administered via the guide cannula.

After an interval of 4 weeks, WM and RM were evaluated once more (Figure 1B). Immediately before the retention test, animals underwent LC stimulation at the abovementioned frequencies, which was followed by the evaluation of WM and RM in the radial maze. The LC stimulation protocols were applied in the same manner as described for Day 1.

### 2.6 Postmortem Verification of Electrode and Cannula Positions

For histological verification of electrodes and cannula positions, brains were prepared for Nissl‐staining. Immediately following euthanasia, cardiac perfusion was conducted with Ringer solution mixed with heparin (0.2%) for 10 min, followed by perfusion with 4% paraformaldehyde solution (PFA) in phosphate‐buffered saline (PBS) at a pH of 7.4 for 15 min. The brain tissue was then stored in 4% PFA for over 1 week, followed by cryoprotection in 30% sucrose solution. Frozen brain sections of 30 μm thickness were obtained using a freezing microtome (Leica CM1325, Leica Microsystems GmbH, Germany) and collected in microplates with 0.1 mL PBS. Then, the sections were mounted on glass slides (SuperFrostPlus, Gerhard Menzel GmbH, Germany) and Nissl‐stained. For this, they were placed in xylene, isopropanol, 96% ethanol, and 70% ethanol for 3 min and then washed in distilled water. The slides were then stained in 0.1% cresyl violet for 3 min. After staining, the slides were washed again in distilled water and further differentiated in 70% ethanol, 96% ethanol, isopropanol, and xylene (3 min in each substance). Mounting was carried out in a histological mounting medium (DePex, Serva Electrophoresis GmbH, Germany). Microphotographs were taken with a digital video camera system (Visitron Systems, Puchheim) on a Leica DM LB Microscope (Leica Mikrosystem Vetrieb GmbH, Wetzlar, Germany) (Figure 1B). All electrode localizations were verified (Figure S2). Animals with incorrect placements were not included in the data analysis.

### 2.7 Data Analysis

Data obtained in the eight‐arm radial maze were acquired on the basis of the number of arm entries, together with the time spent in the maze. Two types of errors were scored depending on the arm entries: RM errors (entry into an unbaited arm or entry into a baited arm without removing the food pellets), or WM errors (re‐entry into a baited arm from which the food pellet had already been collected within that trial) (Naie and Manahan‐Vaughan 2004). All data sets were tested with the D'Agostino and Pearson test of normal distribution (D'Agostino 1986).

A two‐way analysis of variance (ANOVA) was used to evaluate differences in memory (RM errors, WM errors) where “Group” served as a between‐group factor, and the testing day served as the within‐group factor. Here, the difference in performance between days (the last training day versus the experimental day with LC stimulation; the last day before the retention period (Day 6) versus the day after the retention period concluded (Day 34)), was assessed. Data were then subjected to a Sidak's multiple comparison test. A one‐way ANOVA with repeated measures was performed to evaluate the effect of the different experimental conditions, whereby the days of testing/treatment comprised a repeated‐measures factor (factor “treatment”) and performance in the same animals was assessed across different testing/treatment days. Data were then subjected to Tukey's multiple comparison test. The level of significance was set to *p* < 0.05. “Eta squared” was used to measure the effect size in two‐way ANOVA. This reports the proportion of total variance associated with the interaction effect and the main effect. These values are computed by dividing the sum‐of‐squares from the ANOVA table by the total sum‐of‐squares.

Data were analyzed with Statistica software (Version10, TIBCO Software Inc.) and visualized with Prism software (Version 9.1.0 (216), GraphPad Software Inc., USA).

## 3 Results

### 3.1 Phasic Locus Coeruleus Stimulation Before Recent Memory Testing Impairs Recently Acquired Reference Memory

To investigate the role of the LC in memory retrieval, we tested the effect of LC stimulation at frequencies that reflected tonic or phasic activity (2, 5, 10, 20, and 100 Hz).

Animals received LC stimulation immediately before recent memory testing in the eight‐arm radial maze. After 15 days of training (Days −15 through −1, Figure 1C), memory was tested on Day 1.

**FIGURE 2:**
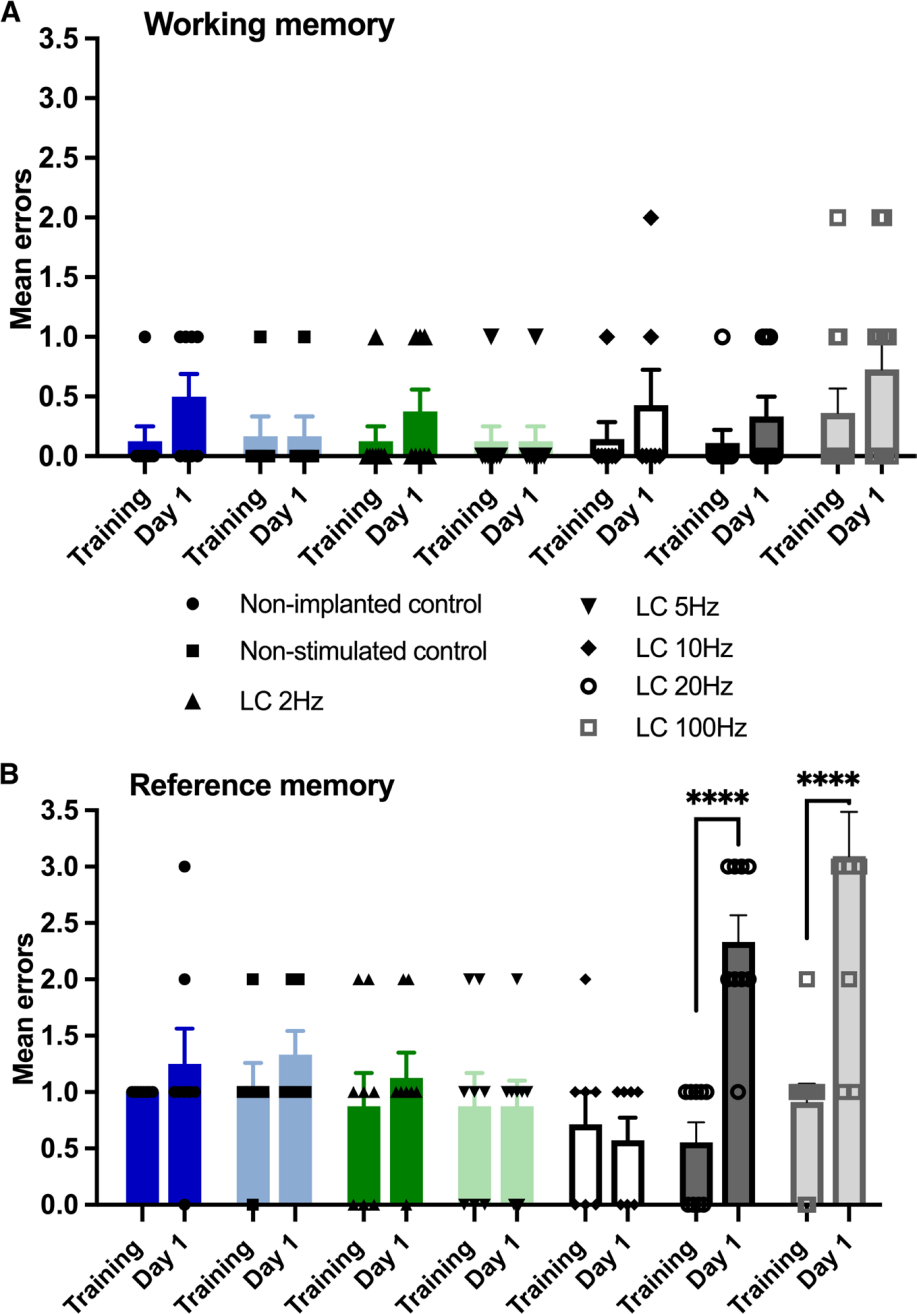
Locus coeruleus stimulation at 20 and 100 Hz, but not 2–10 Hz, impairs retrieval of recently acquired reference memory. Working memory is unaffected. (A) Working memory (WM) performance. No significant differences were found between WM errors after LC stimulation on Day 1 at any of the frequencies tested (2, 5, 10, 20, and 100 Hz), compared with performance on the final acquisition training day (Training). (B) Reference memory (RM) performance. Animals that received LC stimulation at 20 Hz on Day 1 (LC 20 Hz) made significantly more RM errors, compared with performance on the final acquisition training day (Training vs. Day 1, *n* = 9). Animals that received LC stimulation at high‐phasic frequency (LC 100 Hz) also exhibited RM deficits (Training vs. Day 1, *n* = 11). Animals from other stimulated groups (LC 2 Hz, LC 5 Hz and LC 10 Hz) showed no significant increase in RM errors. Significant differences denoted with asterisks; *p < 0.05, **p < 0.01, ***p < 0.001, ****p < 0.0001 (Sidak). Error bars represent ± SEM.

We found that LC stimulation on Day 1 had no effect on WM, regardless of the frequency tested. In particular, there was no significant difference in WM errors between the final acquisition training day (Day −1) and Day 1, when LC stimulation was performed (Figure 2A; Table 1a). By contrast, the effect of LC stimulation on RM was frequency‐dependent: LC stimulation at frequencies of 2–10 Hz had no effect on RM (Figure 2B; Table 1b), while LC stimulation at 20 or 100 Hz potently increased RM errors compared with the final acquisition training day (Figure 2B; Table 1b; Day −1 vs. Day 1 (LC 20 Hz), ****p < 0.0001; Day −1 vs. Day 1 (LC 100 Hz), ****p < 0.0001). These data indicate that LC stimulation at high frequencies (20 and 100 Hz) leads to impairment of the retrieval of recently acquired RM, while having no effect on WM.

**TABLE 1:** Overview of statistical assessment of recent memory performance after LC stimulation at different frequencies compared with the last training day.

a.	Working memory errors	
Two‐way ANOVA, factor Group: *F*(6,50) = 1.07; *p* = 0.38; factor LC stimulation × Group: *F*(6,50) = 0.45; *p* = 0.84; factor LC stimulation: *F*(1,50) = 6.04; *p = 0.02; factor “Group”: *η* ^2^ = 0.07; factor “LC stimulation × Group”: *η* ^2^ = 0.02; factor “LC stimulation”: *η* ^2^ = 0.40		
Group	*p*	*n*
Day‐1 vs. Day 1 (LC 100 Hz) (Sidak)	*p* = 0.38	11
Day‐1 vs. Day 1 (LC 20 Hz) (Sidak)	*p* = 0.92	9
Day‐1 vs. Day 1 (LC 10 Hz) (Sidak)	*p* = 0.86	6
Day‐1 vs. Day 1 (LC 5 Hz) (Sidak)	*p* = 0.99	7
Day‐1 vs. Day 1 (LC 2 Hz) (Sidak)	*p* = 0.89	7
Day‐1 vs. Day 1 (Non‐stimulated control) (Sidak)	*p* = 0.99	6
Day‐1 vs. Day 1 (Non‐implanted control) (Sidak)	*p* = 0.54	8

b.	Reference memory errors	
Two‐way ANOVA, factor “Group”: *F*(6,50) = 4.86; ***p = 0.0006; factor “LC stimulation × group”: *F*(6,50) = 10.79; ****p < 0.0001; factor “LC stimulation”: *F*(1,50) = 33.52; *p* < 0.0001; factor “Group”: *η* ^2^ = 0.21; factor “LC stimulation × Group”: *η* ^2^ = 0.24; factor “LC stimulation”: *η* ^2^ = 0.12		
Group	*p*	*n*
Day‐1 vs. Day 1 (LC 100 Hz) (Sidak)	****p < 0.0001	11
Day‐1 vs. Day 1 (LC 20 Hz) (Sidak)	****p < 0.0001	9
Day‐1 vs. Day 1 (LC 10 Hz) (Sidak)	*p* = 0.99	6
Day‐1 vs. Day 1 (LC 5 Hz) (Sidak)	*p* = 0.99	7
Day‐1 vs. Day 1 (LC 2 Hz) (Sidak)	*p* = 0.97	7
Day‐1 vs. Day 1 (Non‐stimulated control) (Sidak)	*p* = 0.94	6
Day‐1 vs. Day 1 (Non‐implanted control) (Sidak)	*p* = 0.97	8

*Note:* In each case working or reference memory errors were assessed immediately after application of LC stimulation protocols in the frequency range of 2–100 Hz. The two control groups (non‐stimulated controls, non‐implanted controls) followed identical behavioral testing in the absence of stimulation (see Section 2). Performance was compared against errors detected on the final acquisition training day (Training). Panels report: (a) Working memory errors. (b) Reference memory errors. Asterices denote statistical significance: *p < 0.05, **p < 0.01, ***p < 0.001, ****p < 0.0001.

Abbreviations: ANOVA, analysis of variance; LC, locus coeruleus.

### 3.2 Reference Memory Impairment Caused by Phasic LC Stimulation is Prevented by Pretreatment With a β‐Adrenergic but Not a Dopamine D1/5 Receptor Antagonist

**FIGURE 3:**
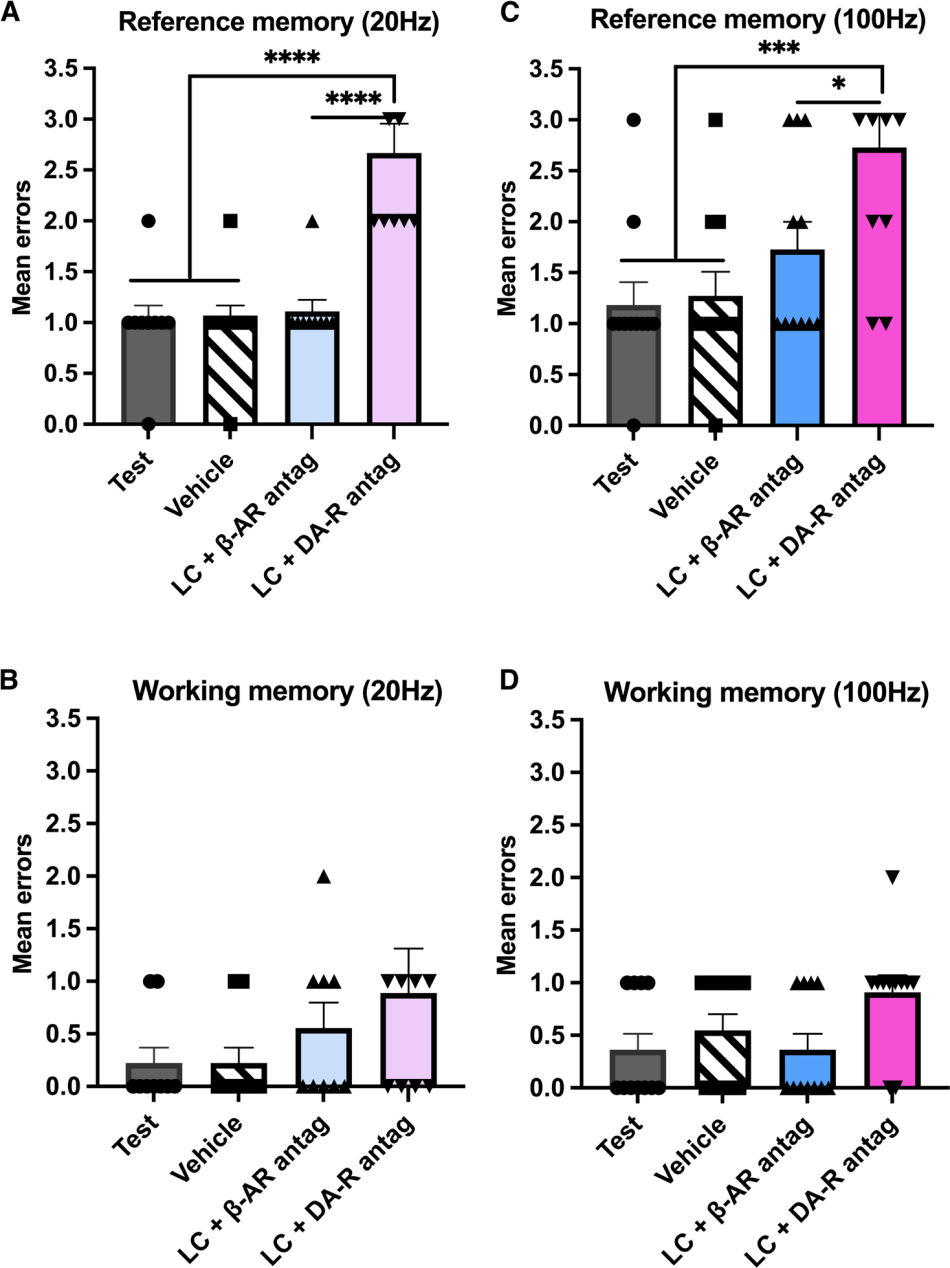
Frequency‐dependence of prevention by beta‐adrenergic receptor antagonists of the increase in reference memory errors caused by LC stimulation before retrieval. Working memory is unaffected. Reference memory (RM) performance after LC stimulation at 20 Hz. Administration of a β‐AR antagonist (antag) before LC stimulation at 20 Hz prevented the increase in RM errors (Test vs. LC 20 Hz + β‐AR antagonist, *n* = 9). By contrast, pretreatment with a D1/D5R antagonist had no effect on RM performance (Test vs. LC 20 Hz + DA‐R antagonist, *n* = 9). There was also a significant difference between the effect of a β‐AR‐antagonist compared with the effect of a D1/D5R antagonist on RM deficits elicited by LC stimulation at 20 Hz (LC 20 Hz + β‐AR antagonist vs. LC 20 Hz + D1/D5R antagonist, *n* = 9). (B) Working memory (WM) performance after LC stimulation at 20 Hz. Administration of either a β‐AR antagonist, or D1/D5R antagonist, before LC stimulation at 20 Hz had no significant effect on WM errors (Test vs. LC 20 Hz + β‐AR‐antagonist, *n* = 9; Test vs. LC 20 Hz + D1/D5R antagonist, *n* = 9). (C) RM performance after LC stimulation at 100 Hz. Pretreatment with a β‐AR antagonist before LC stimulation at 100 Hz significantly decreased RM errors (Test vs. LC 100 Hz + β‐AR antag, *n* = 11). By contrast, D1/D5R antagonism did not prevent the increase in RM errors caused by LC stimulation at 100 Hz (Test vs. LC 20 Hz + D1/D5R antag, *n* = 11). The reduction by β‐AR antagonism of RM errors (following LC stimulation) was significantly different from the effect of the D1/D5R antagonism on RM (LC 100 Hz + β‐AR antagonist vs. LC 100 Hz + D1/D5R antag; *n* = 11). (D) WM performance after LC stimulation at 100 Hz. Administration of either a β‐AR antagonist, or a D1/D5R antagonist, before LC stimulation at 100 Hz, had no significant effect on WM errors (Test vs. LC 100 Hz + β‐AR‐antagonist, *n* = 11; Test vs. LC 100 Hz + D1/D5R antagonist, *n* = 11). Significant differences denoted with asterisks *p < 0.05, ** *p* < 0.01, *** *p* < 0.001, **** *p* < 0.0001 (Tukey). Error bars represent ± SEM.

Given the abovementioned finding that LC stimulation, at frequencies akin to phasic and high‐phasic activity, caused an impairment of recently acquired RM, we explored the involvement of NA or dopamine (DA) in mediation of this memory deficit. To test this, before LC stimulation at 100 Hz or at 20 Hz, animals were treated with either a β‐AR antagonist or a D1/D5R antagonist. We observed that the deficit in RM retrieval caused by LC stimulation at 20 Hz was prevented by the administration of the β‐AR antagonist, propranolol: there was no significant difference between Day 2, when RM was initially evaluated, and RM on Day 4 when LC stimulation was combined with β‐AR antagonist treatment (Figure 3A; Table 2a). By contrast, we found no effect on RM that was elicited by the D1/D5R antagonist, SCH23390, on RM. In this case, the application of the D1/D5R antagonist did not prevent the increase in RM errors caused by LC stimulation at 20 Hz (Figure 3A; Table 2a; memory test vs. LC 20 Hz + D1/D5R antagonist, ****p < 0.0001). Furthermore, the rescue of RM errors by treatment with the β‐AR antagonist was significantly higher than the responses registered in the presence of the D1/D5R antagonist (Figure 3A; Table 2a; LC 20 Hz + β‐AR antagonist vs. LC 20 Hz + D1/D5R antagonist, ****p < 0.0001). Neither β‐AR antagonist nor D1/D5R antagonist treatment affected WM (Figure 3B; Table 2b).

**TABLE 2:** Overview of statistical assessment of the effect of β‐adrenergic receptor and D1/D5 receptor antagonist treatment on impairment of recent memory induced by LC stimulation at 20 Hz and at 100 Hz.

a.	Reference memory errors (LC stimulated at 20 Hz)	
One‐way rmANOVA, *F*(3,24) = 26.18; ****p < 0.0001		
Treatment conditions	*p*	*n*
Memory test vs. vehicle (Tukey)	*p* = 0.99	9
Memory test vs. LC 20 Hz + β‐AR antagonist (Tukey)	*p* = 0.96	9
Memory test vs. LC 20 Hz + D1/D5R antagonist (Tukey)	****p < 0.0001	9
Vehicle vs. LC 20 Hz + β‐AR antagonist (Tukey)	*p* = 0.96	9
Vehicle vs. LC 20 Hz + D1/D5R antagonist (Tukey)	****p < 0.0001	9
LC 20 Hz + β‐AR antagonist vs. LC 20 Hz + D1/D5R antagonist (Tukey)	****p < 0.0001	9

b.	Working memory errors (LC stimulated at 20 Hz)	
One‐way rmANOVA, *F*(3,24) = 1.42; *p* = 0.26		
Treatment conditions	*p*	*n*
Memory test vs. vehicle (Tukey)	*p* = 0.99	9
Memory test vs. LC 20 Hz + β‐AR antagonist (Tukey)	*p* = 0.82	9
Memory test vs. LC 20 Hz + D1/D5R antagonist (Tukey)	*p* = 0.32	9
Vehicle vs. LC 20 Hz + β‐AR antagonist (Tukey)	*p* = 0.82	9
Vehicle vs. LC 20 Hz + D1/D5R antagonist (Tukey)	*p* = 0.32	9
LC 20 Hz + β‐AR antagonist vs. LC 20 Hz + D1/D5R antagonist (Tukey)	*p* = 0.82	9

c.	Reference memory errors (LC stimulated at 100 Hz)	
One‐way rmANOVA, *F*(3,30) = 10.37; ****p < 0.0001		
Treatment conditions	*p*	*n*
Memory test vs. vehicle (Tukey)	*p* = 0.99	11
Memory test vs. LC 100 Hz + β‐AR antagonist (Tukey)	*p* = 0.31	11
Memory test vs. LC 100 Hz + D1/D5R antagonist (Tukey)	****p < 0.0001	11
Vehicle vs. LC 100 Hz + β‐AR antagonist (Tukey)	*p* = 0.47	11
Vehicle vs. LC 100 Hz + D1/D5R antagonist (Tukey)	***p < 0.0003	11
LC 100 Hz + β‐AR antagonist vs. LC 100 Hz + D1/D5R antagonist (Tukey)	*p < 0.02	11

d.	Working memory errors (LC stimulated at 100 Hz)	
One‐way rmANOVA, *F*(3,30) = 2.62; *p* = 0.07		
Treatment conditions	*p*	*n*
Memory test vs. vehicle (Tukey)	*p* = 0.84	11
Memory test vs. LC 100 Hz + β‐AR antagonist (Tukey)	*p* = 0.99	11
Memory test vs. LC 100 Hz + D1/D5R antagonist (Tukey)	*p* = 0.093	11
Vehicle vs. LC 100 Hz + β‐AR antagonist (Tukey)	*p* = 0.84	11
Vehicle vs. LC 100 Hz + D1/D5R antagonist (Tukey)	*p* = 0.38	11
LC 100 Hz + β‐AR antagonist vs. LC 100 Hz + D1/D5R antagonist (Tukey)	*p* = 0.093	11

*Note:* β‐AR antagonist, D1/D5R antagonist, or vehicle, were injected 30 min before application of the LC stimulation protocol, followed by memory assessment in the radial maze. Panels report: (a) Reference memory errors detected after LC stimulation at 20 Hz under the different treatment conditions. (b) Working memory errors detected after LC stimulation at 20 Hz under the different treatment conditions. (c) Reference memory errors detected after LC stimulation at 100 Hz under the different treatment conditions. (d) Working memory errors detected after LC stimulation at 100 Hz under the different treatment conditions. Asterices denote statistical significance: *p < 0.05, **p < 0.01, ***p < 0.001, ****p < 0.0001.

Abbreviations: LC, locus coeruleus; rmANOVA, analysis of variance with repeated measures (rmANOVA).

Then, we examined whether the effect of LC stimulation at 100 Hz on memory retrieval depends on β‐AR or D1/D5R. We found that RM deficits caused by 100 Hz were prevented by pretreatment with the β‐AR antagonist: the number of RM errors was not significantly different compared with RM assessment detected on Day 2 (Figure 3C; Table 2c). By contrast, pretreatment with the D1/D5R antagonist had no effect on RM errors induced by LC stimulation at 100 Hz (Figure 3C; Table 2c; memory test vs. LC 100 Hz + D1/D5R antagonist; ****p < 0.0001). The effect of β‐AR antagonism on RM was significantly different from the effect of D1/D5R antagonism on RM deficits elicited by LC stimulation at 100 Hz (Figure 3C; Table 2c; LC 100 Hz + β‐AR antagonist vs. LC 100 Hz + D1/D5R antagonist; *p < 0.02). This suggests that deficits in RM caused by LC stimulation, at phasic and high phasic frequencies, depend on NA acting on β‐AR.

With regard to WM, pretreatment with the β‐AR or D1/D5R antagonists had no significant effects (Figure 3D; Table 2d).

### 3.3 LC Activation Before Remote Memory Retrieval Reduces Working Memory Errors

**FIGURE 4:**
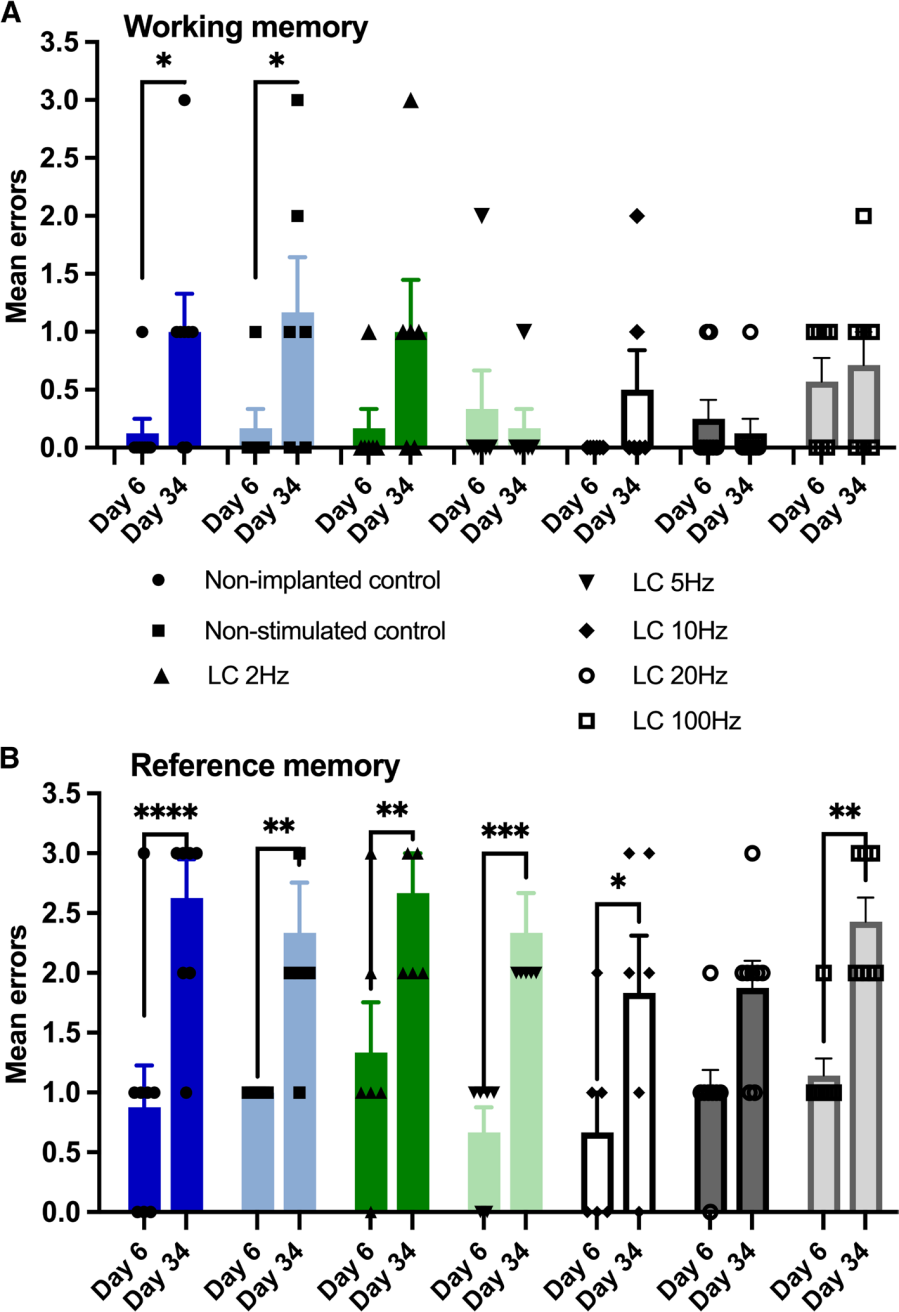
Working memory is impoverished in controls after 4 weeks and rescued by locus coeruleus stimulation at both low and high frequencies. Locus coeruleus stimulation does not affect retrieval of reference memory acquired 4 weeks previously. (A) Working memory (WM) performance after the retention period. In both control groups (Non‐stimulated control, *n* = 6; Non‐implanted control, *n* = 8), significantly higher errors were detected in WM performance compared with performance detected 4 weeks previously (Day 6) (Day 6 vs. Day 34, all *p < 0.05). By contrast, LC stimulation at any tested frequency (LC 2 Hz, LC 5 Hz, LC 10 Hz, LC 20 Hz, LC 100 Hz) before testing WM, resulted in error levels that were equivalent to those detected 4 weeks previously (Day 6), suggesting that LC stimulation improves WM performance (Day 6 vs. Day 34). (B) Reference memory (RM) performance after the retention period. In controls, when RM was tested 4 weeks after the final test during the previous trials, errors were significantly higher (Day 6 vs. Day 34, Non‐stimulated control: *n* = 6; Non‐implanted control: *n* = 8). LC stimulation had no effect on RM (Day 6 vs. Day 34) Significant differences denoted with asterisks *p < 0.05, ** *p* < 0.01, *** *p* < 0.001, **** *p* < 0.0001 (Sidak). Error bars represent ± SEM.

Four weeks after the conclusion of the abovementioned tests, that is on Day 34, we explored to what extent LC stimulation immediately before remote memory testing affects WM and RM. The animals received LC stimulation immediately before the retention test in the radial maze, as was done on Day 1. Two control groups (electrodes present in LC, electrodes absent) did not receive stimulation but followed the same procedures as the stimulated animals. RM and WM performance was compared with performance on Day 6, when memory was tested *before* the 4‐week retention period. We found that both groups of control animals made significantly more RM errors on Day 34 compared with Day 6 (Figure 4B, Table 3b). Stimulation of the LC at any tested frequency did not significantly affect RM performance on Day 34 compared with Day 6 (Figure 4B, Table 3b).

**TABLE 3:** Overview of statistical assessment of remote memory performance after LC stimulation at different frequencies compared with performance on the last day before the retention period.

a.	Working memory errors	
Two‐way ANOVA, factor “Group”: *F*(6,40) = 1.047; *p* = 0.41; factor “LC stimulation × Group”: *F*(6,40) = 2.33; *p* = 0.50; factor “LC stimulation”: *F*(1,40) = 12.72; ***p = 0.001; factor “Group”: *η* ^2^ = 0.07; factor “LC stimulation × Group”: *η* ^2^ = 0.09; factor “LC stimulation”: *η* ^2^ = 0.09		
Group	*p*	*n*
Day 6 vs. Day 34 (LC 100 Hz) (Sidak)	*p* = 0.99	8
Day 6 vs. Day 34 (LC 20 Hz) (Sidak)	*p* = 0.99	8
Day 6 vs. Day 34 (LC 10 Hz) (Sidak)	*p* = 0.67	6
Day 6 vs. Day 34 (LC 5 Hz) (Sidak)	*p* = 0.99	6
Day 6 vs. Day 34 (LC 2 Hz) (Sidak)	*p* = 0.12	6
Day 6 vs. Day 34 (Non‐stimulated control) (Sidak)	*p < 0.05	6
Day 6 vs. Day 34 (Non‐implanted control) (Sidak)	*p < 0.05	8

b.	Reference memory errors	
Two‐way ANOVA, factor “Group”: *F*(6,40) = 0.96; *p* = 0,46; factor “LC stimulation × Group”: *F*(6,40) = 0.80; *p* = 0.57; factor “LC stimulation”: *F*(1,40) = 102.4; ****p < 0.0001; factor “Group”: *η* ^2^ = 0.04; factor “LC stimulation × Group”: *η* ^2^ = 0.02; factor “LC stimulation”: *η* ^2^ = 0.43.		
Group	*p*	*n*
Day 6 vs. Day 34 (LC 100 Hz) (Sidak)	**p < 0.0037	8
Day 6 vs. Day 34 (LC 20 Hz) (Sidak)	*p* = 0.06	8
Day 6 vs. Day 34 (LC 10 Hz) (Sidak)	*p < 0.02	6
Day 6 vs. Day 34 (LC 5 Hz) (Sidak)	***p < 0.0004	6
Day 6 vs. Day 34 (LC 2 Hz) (Sidak)	**p < 0.005	6
Day 6 vs. Day 34 (Non‐stimulated control) (Sidak)	**p < 0.005	6
Day 6 vs. Day 34 (Non‐implanted control) (Sidak)	****p < 0.0001	8

*Note:* A memory retention test was conducted on Day 34, which amounted to 28 days after the last exposure to the radial maze that occurred on Day 6. Working and reference memory errors were then assessed immediately after application of LC stimulation protocols in the frequency range of 2–100 Hz. The two control groups (non‐stimulated controls, non‐implanted controls) followed identical behavioral testing in the absence of stimulation (see methods section). Performance was compared against errors detected on the last day before the retention period (Day 6). Panels report: (a) Working memory errors. (b) Reference memory errors. Asterices denote statistical significance: *p < 0.05, **p < 0.01, ***p < 0.001, ****p < 0.0001.

Abbreviations: ANOVA, analysis of variance; LC, locus coeruleus.

We did, however, observe that LC stimulation improved WM on Day 34 (compared with Day 6), as was reflected by the absence of significant differences in WM errors at all frequencies tested (Figure 4A, Table 3a). By contrast, both control groups made significantly more WM errors than on Day 34 (Figure 4A, Table 3a; Non‐stimulated control vs. Day 6, *p < 0.05; Non‐implanted control vs. Day 6, *p < 0.05).

These data indicate that LC activation in phasic ranges elicits distinctly different outcomes in spatial memory performance, depending on whether this occurs during the retrieval of recently acquired or remotely acquired memories.

## 4 Discussion

In this study, the role of LC in memory retrieval was investigated in a memory spatial task that included both WM and RM components. For this, spatial memory in an eight‐arm radial maze was examined. This task requires that rats learn two strategies: first, they must learn that only four of the 8 arms contain food rewards and that these arms are always the same from day to day (RM). Second, they must learn that when a food reward is removed, it will not be replaced and thus, it makes no sense to return to an arm from which a reward was retrieved on a given day (WM). We were particularly interested in ascertaining to what extent activation of the LC influences memory retrieval *after* the animals had learned the tasks well. For this, we explored whether the influence of the LC on memory retrieval is frequency‐dependent and whether it differs depending on whether LC activation occurs immediately before retrieval of a recently acquired memory, compared with retrieval of spatial memory that had been acquired 4 weeks previously.

We detected differences in the effect of LC activation on the retrieval of recently acquired memories, compared with the retrieval of remote memories, whereby effects on WM or RM could be differentiated. Moreover, the effect of LC activation on memory was frequency‐dependent: LC stimulation in the range of phasic, but not tonic, frequencies, before the retrieval of recently acquired memory, caused deficits in RM, but not WM. Effects were β‐AR dependent. Recent memory testing 1 day later, in the absence of any manipulation, revealed normal RM, suggesting that the disruption was attention‐based and did not alter consolidated memory. By contrast, LC stimulation after a long retention period of 28 days exerted a facilitatory effect on WM: LC stimulation at 2–100 Hz led to the improvement of WM on Day 34 compared with Day 6 of testing. These findings support the interpretation that LC modulation of memory retrieval is not only frequency‐dependent, but also state‐dependent. LC stimulation may also have directly influenced the perception of the contextual context by the animals during the retrieval of recent and remote RM. Dekeyne and colleagues demonstrated that a 90 s detainment of rats in a wire‐mesh cage, next to a maze in which RM was acquired, was an effective reminder of maze learning and facilitated memory retrieval after a long retention period (Dekeyne et al. 1987). Thus, LC stimulation may have also promoted the perception of the maze context.

LC neurons exhibit two distinct firing modes: tonic and phasic (Aston‐Jones and Cohen 2005). Tonic activity is associated with a spontaneous fluctuation across the low‐frequency range, which co‐varies with arousal levels. Low‐tonic LC activity (1–2 Hz) is positively correlated with the arousal state (Aston‐Jones and Bloom 1981a), whereas high‐tonic LC activity (3–8 Hz) is observed during stressful events (Curtis et al. 2012; Snyder et al. 2012), exploratory behavior, and labile attention that is associated with decreased task utility. High‐tonic LC activity also occurs during changes in behavior for the purpose of searching for an alternative behaviorally beneficial outcome (Aston‐Jones and Cohen 2005) which, in turn, favors behavioral flexibility (Aston‐Jones et al. 2007). By contrast, phasic burst activity is associated with focusing attention and maintaining engagement in an ongoing behavioral task (Clayton et al. 2004). This kind of activity is characterized by 2–4 LC spikes at 10–25 Hz, which are prompted by salient or novel stimuli or decision signals from prefrontal cortical regions (Aston‐Jones and Bloom 1981b; Vankov et al. 1995; Takeuchi et al. 2016). Moreover, high phasic activity in the range of 100 Hz occurs in response to vagal nerve stimulation and may thus reflect periods of high arousal (Hulsey et al. 2017).

Overall, the stimulation protocols we used were based on studies by others of single‐cell and multi‐cell activity in the LC and were selected to mimic approximated (averaged) physiological discharges of individual LC neurons in different conditions. For example, ex vivo intracellular brain slice recordings have demonstrated spontaneous firing rate variability among LC neurons (Williams et al. 1984). Thus, the frequency of the stimulation protocol was aimed at emulating average LC firing during particular conditions that would comprise the pooling of individual LC neuronal responses of varying frequencies into a population output. The tonic stimulation protocol we used, comprising 2 Hz for 20 s, is consistent with LC activity recorded during the waking state (Aston‐Jones and Bloom 1981a). Tonic stimulation at 5 Hz for 20 s was chosen to mimic stress‐related activity (Curtis et al. 2012; McCall et al. 2015). LC cells exhibit phasic bursts of activity (2–3 spikes; 10–20 Hz) in response to salient sensory stimuli and during decision‐making processes (Aston‐Jones and Bloom 1981a; Clayton et al. 2004; Aston‐Jones and Cohen 2005). The phasic stimulation protocol at 20 Hz was therefore chosen to mimic the activity of LC neurons in response to novel sensory stimuli (Takeuchi et al. 2016). Moreover, the same stimulation protocol was found to facilitate memory retrieval (Sara and Devauges 1988). The phasic stimulation protocol at 100 Hz was previously used in an episodic‐like memory task and caused optimization of “what–where–when” memory (Lemon et al. 2009). Moreover, in another study, it was reported that LC stimulation at 100 Hz leads to a reference memory deficit (Novitskaya et al. 2016). We are aware of reports that frequency‐dependent synchrony can be instigated (in a rat slice preparation) by increasing the correlation of the membrane potential between pairs of neurons (Alvarez et al. 2022). By contrast, increasing the firing frequency decreased the amplitude and synchrony of the oscillations among pairs, leading to desynchronization (Alvarez et al. 2022). Others have shown, however, in an in vivo experiment, that LC multi‐unit activity, in the form of excitation followed by inhibition lasting 500 ms to 1 s, occurred after a paw pinch (Cedarbaum and Aghajanian 1978). Thus, we believe that the protocols used for phasic stimulation described above are justified.

We observed that stimulation of the LC at frequencies similar to tonic (2 or 5 Hz) or low‐phasic activity of the LC (10 Hz) has no effect on RM or WM of a recently acquired radial maze task. By contrast, LC stimulation at 20 or 100 Hz, akin to phasic and high‐phasic LC activity (Aston‐Jones and Bloom 1981b; Hulsey et al. 2017), impairs RM while leaving WM intact. The latter finding suggests that high‐frequency activity of the LC does not disrupt *ongoing* attention. Although RM was disrupted immediately after LC stimulation, it had returned to criterion levels on the day after LC stimulation. This indicates that the integrity of long‐term memory was not impaired by high‐frequency LC stimulation: rather, it may have been that the outcome of LC activation was that attention to novelty was prioritized over attention to retrieval cues. Another possibility is that high‐frequency stimulation of the LC may have signaled that the animals should update their previously acquired representations. This interpretation is supported by previous reports that LC stimulation at high frequencies results in hippocampal information encoding in the form of synaptic plasticity (Lemon et al. 2009; Babushkina and Manahan‐Vaughan 2022) and that it optimizes “what–where–when memory” that is acquired in a temporal sequence (Lemon et al. 2009).

A large body of evidence suggests that LC activation enhances memory acquisition and consolidation (Lemon et al. 2009; Kempadoo et al. 2016; Wagatsuma et al. 2018; Novitskaya et al. 2016; Takeuchi et al. 2016). In a complex episodic‐like memory task, LC stimulation at a high phasic frequency (100 Hz), immediately before exposure to a spatiotemporal episode, facilitates memory encoding for that episode in a β‐AR‐dependent manner (Lemon et al. 2009). Optogenetic activation of the LC at 20 Hz during training sessions significantly enhances spatial learning in mice and is prevented by injection of a D1/D5R antagonist (Kempadoo et al. 2016). Similar effects have been reported in rats engaging in a flexible choice task during 25 Hz LC optogenetic stimulation (Tse et al. 2023). Furthermore, post‐encoding LC stimulation at 25 Hz enhances memory of an episodic‐like experience for over 24 h in mice (Takeuchi et al. 2016). This suggests that phasic and high‐phasic LC activity may promote the consolidation of recent memory. We found that LC stimulation at these and other frequencies impaired RM, however. The possibility arises that the frequencies applied in the present study occur on the backdrop of intrinsic, state‐dependent LC activity and may be additive. Several studies have demonstrated that LC activation and associated NA transmission are required for the retrieval of spatial memory (Murchison et al. 2004; Korz and Frey 2007; Durán et al. 2023), which indicates that, during the retrieval of recently acquired memory, a certain level of NA transmission is already ongoing. An inverted U‐shaped relationship between arousal and task performance has been proposed, whereby increasing arousal enhances the efficiency of task performance, with the exception of very high levels of arousal, which conversely decrease task performance through promoting anxiety or stress (Easterbrook 1959; Eysenck 1982). Under stressful conditions, a high level of NA is released in the PFC that activates α1‐adrenergic receptors, the activation of which promotes flexible attention (Lapiz and Morilak 2006). This suggests that LC stimulation at phasic (20 Hz) and high‐phasic (100 Hz) frequencies leads to increases in NA concentration on top of already existing levels, which may be counterproductive in the case of memory retrieval. This high frequency of LC activity might rather convey a signal about changes within the environment and produce a state of high behavioral flexibility, or “scanning attention” that prompts the animals to spend more time exploring the maze for potentially new salient cues, rather than focusing their attention on the recently learned task (Aston‐Jones et al. 1999).

When we tested RM and WM 4 weeks after the conclusion of the first training and test series, we found that control animals, as well as animals from the groups which received LC stimulation, exhibited significant increases in their errors in both forms of memory. Only LC stimulation at 20 Hz exhibited a tendency, albeit non‐significant (*p* < 0.06), to facilitate retrieval of RM. Previously it has been shown that the same LC stimulation protocol leads to facilitation of memory of an appetitive linear maze task after a retention period (Sara and Devauges 1988). The less prominent effect of LC stimulation on remote RM that we report here may be related to the spatial complexity of the radial maze task, compared with the linear maze that included procedural components.

Although we observed no effect of LC stimulation on remote RM (Day 34), we observed a significant reduction of WM errors following LC stimulation, compared with control animals on the same testing day. Strikingly, LC stimulation at any of the test frequencies returned WM levels to those detected 4 weeks previously. It is tempting to speculate that, in this case, stimulating the LC served as an attention booster that helped the animals remember from which arm they had already retrieved a reward. But it also raises the question as to why this did not work during WM of the recently acquired task.

One possibility is that, during the retrieval of recently acquired memory, LC stimulation has no effect on WM, because the animals had recently learned well that reward pellets were not replaced within a current trial. This rule related to WM seems to be comparatively easy to learn, given that the rats reached the learning criteria for WM much faster than for RM. In other words, LC activation had no effect on an already superior WM. In line with this interpretation, it has been proposed that LC activity is not critical for the daily performance of well‐learned and well‐remembered tasks (Sara and Devauges 1988). By contrast, 4 weeks after learning the task, LC activation may boost attention to restore WM to past performance levels.

These abovementioned frequencies have an impact on both NA and DA release in the hippocampus or in cortical structures. One study reported frequency‐dependent elevations of NA in the prefrontal cortex, whereby 3 Hz was ineffective and 5–10 Hz produced elevations (Florin‐Lechner et al. 1996). Moreover, phasic activity of LC results in a greater increase in NA levels in the prefrontal cortex compared with tonic stimulation, and the higher the phasic frequency, the more NA is released (Tanaka et al. 1976; Florin‐Lechner et al. 1996). Furthermore, LC stimulation of rats at 100 Hz results in a potent increase in NA and a smaller increase in DA levels in the hippocampal CA1 region (Lemon et al. 2009). In addition, it has been shown that hippocampal long‐term depression (LTD), which occurs when test‐pulse stimulation of hippocampal afferents is implemented during LC stimulation, exhibits a frequency dependency of the recruitment of D1/D5 receptors or β‐AR, whereby lower LC frequencies enable LTD that depends on activation of D1/D5 receptors, and higher phasic frequencies promote β‐AR‐dependent hippocampal LTD (Babushkina and Manahan‐Vaughan 2022).

Our results clearly show that state‐dependent activity of the LC exerts differential effects on the retrieval of recent and remote memory. The question arises as to the locus of these processes. Retention of recent spatial discrimination memory by mice is accompanied by increased neuronal activity in the dorsal hippocampus (CA1, CA3, and dentate gyrus), along with cortical areas such as parietal cortex and posterior cingulate cortex (Bontempi et al. 1999). Retention of remote memory of the same experience is characterized by decreased metabolic activity in the dorsal hippocampus and increased activity in several cortical areas, including the prefrontal and anterior cingulate cortices, as well as the parietal cortex. More recently, it was reported that the dentate gyrus and cornus ammonis are needed for recent memory retrieval (Carretero‐Guillén et al. 2024). These findings are supported by studies of immediate early gene expression occurring during associative learning and detected during memory retrieval (Frankland et al. 2004; Maviel et al. 2004). In addition to our findings, others have provided evidence that LC‐hippocampal communication is involved in these processes: infusion of a β‐AR agonist into the dentate gyrus before retrieval causes deficits of reference memory acquired in a radial maze (Grella et al. 2021). However, antagonism of β‐AR or D1/5R prevents spatial context learning and most forms of persistent hippocampal synaptic plasticity (Hansen and Manahan‐Vaughan 2014; Hagena et al. 2016). A core engram of learned experience may be retained in the hippocampus (Rashid et al. 2023). Nonetheless, given that memory retention over longer periods of time becomes more dependent on the neocortex, the facilitation effect of LC stimulation on remote memory might be explained by the involvement of LC–prefrontal cortex projections in the modulation of attention (McGaughy et al. 2008), as well as by direct effects of LC activity on the hippocampus. Moreover, NA release from the LC to the medial prefrontal cortex during memory encoding is critical for engram tagging in the prefrontal cortex, as well as for remote memory storage (Kitamura et al. 2017; Matos et al. 2018; Fan et al. 2022).

In light of contemporary knowledge derived from mouse and rat studies that the LC releases both NA and DA (Smith and Greene 2012; Lemon and Manahan‐Vaughan 2012; Kempadoo et al. 2016; Takeuchi et al. 2016) we explored whether those frequencies that exhibited the most potent effect on RM (20 and 100 Hz) resulted in the recruitment of β‐AR during the retrieval of recently acquired memory. We found that RM impairments caused by LC stimulation at 20 Hz and at 100 Hz were β‐AR and not D1/5R‐dependent, in contrast to findings that DA release from the LC (following optogenetic photostimulation at 25 Hz) supports the stabilization of memory about environmental novelty (Takeuchi et al. 2016). This suggests that the contribution of the LC to memory processes differs, depending on the memory phase and/or component under scrutiny, and depending on LC activity patterns. Moreover, although DA and NA both play an important role in both memory acquisition (Lemon et al. 2009; Kempadoo et al. 2016; Wagatsuma et al. 2018; Tsetsenis et al. 2022) and consolidation (Bevilaqua et al. 1997; Sara et al. 1999; Ji et al. 2003; Durán et al. 2023; Takeuchi et al. 2016; Tse et al. 2023), their impact on memory retrieval is not equal. NA release is required for the retrieval of different types of hippocampus‐dependent memory (Devauges and Sara 1991; Murchison et al. 2004; Korz and Frey 2007; Durán et al. 2023). Information about the contribution of DA in memory retrieval is not consistent, however. For example, D1/5R antagonism targeting the dorsal hippocampus or several cortical areas, implemented shortly before behavioral testing, impairs inhibitory avoidance memory, while D1/5R receptor agonism before a 24‐h retention test enhances memory retrieval (Barros et al. 2001). By contrast, several studies have shown that D1/5R antagonism before a memory retention test in rodents has no effect on the retrieval of fear conditioning memory or spatial memory (Inoue et al. 2000; O'Carroll et al. 2006). Other studies support the view that DA is mainly required for the persistence of synaptic plasticity and memory consolidation (Huang and Kandel 1995; Sajikumar and Frey 2004; Lemon and Manahan‐Vaughan 2006). Thus, whereas *both* NA and DA release from the LC might work in a complementary manner to gate new information into long‐term storage (Hagena and Manahan‐Vaughan 2025), in the case of memory retrieval, the role of NA release from the LC appears to be more prominent.

## 5 Conclusions

Our results indicate that LC stimulation has different effects on memory retrieval depending on the frequency of LC stimulation and the latency between learning and retrieval. In the case of well‐consolidated memory, LC stimulation in the range of phasic frequencies transiently disrupts memory retrieval, whereas when the latency between learning and retrieval is extended, LC activation facilitates memory retrieval, possibly via shifting attention toward the contextual cues associated with the learning task. Moreover, our study revealed that the primary neuromodulator that supports the retrieval of spatial memory by the LC is NA.

## Author Contributions

The study was designed by D.M.‐V. Experiments were conducted by N.B. and analyzed by N.B. and D.M.‐V. The article was written by both authors.

## Conflicts of Interest

The authors declare no conflicts of interest.

## Supporting information


**Figure S1.** Performance across 15 days of the experiment.

**Figure S2.** Histological verification and reconstruction of the electrode placement in LC.

## Data Availability

The data from this study are available from the corresponding author upon reasonable request.
