# Supplementary material for: The Modulation by the Locus Coeruleus of Recent and Remote Memory Retrieval is Activity‐Dependent
Source: Hippocampus. 2025 Feb 20;35(2):e70004. doi: 10.1002/hipo.70004 (PMC11842585; doi:10.1002/hipo.70004)
Supplement: Supplementary file 1 — Figure S1. Performance across 15 days of the experiment. [file HIPO-35-0-s002.pdf]

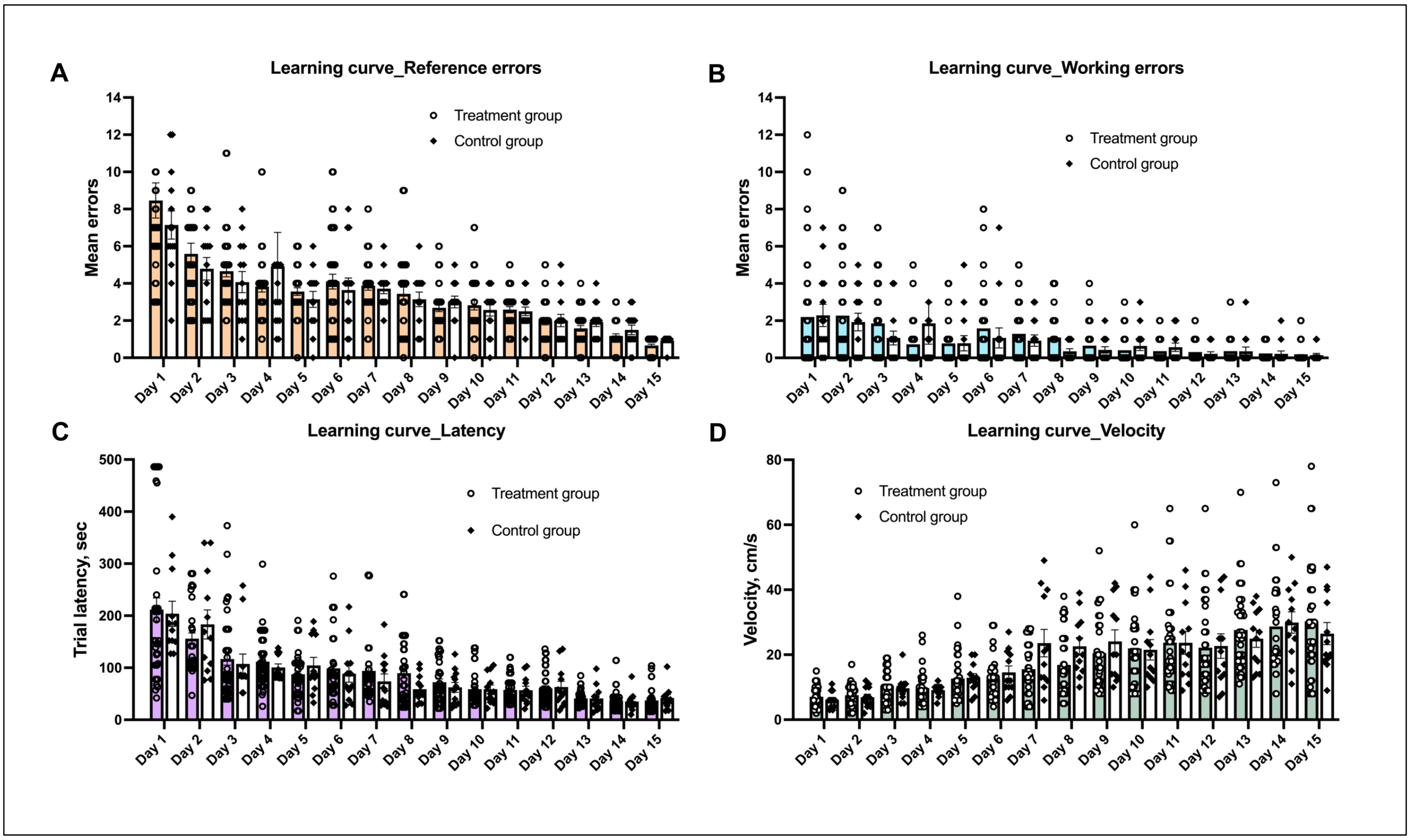

Supplementary Figure 1. Performance across 15 days of the experiment

(A) Learning curve for mean Reference memory errors.
(B) Learning curve for mean Working memory errors.
(C) Latency (s), represents the time taken to collect all reward pellets. Animals were considered to have learnt the task when mean working and reference memory errors were < 1 per trial.
(D) Velocity (cm/s) represents animal locomotion , as determined by a calculation based on the amount of time spent in the radial maze and the number of arms entered: (number of arms entered × 160)/(time spent in maze (s), whereby 16 comprises the length of the maze from arm tip to opposite arm tip in cm.
The animals were randomly sorted into either control groups (excepting rats that did not undergo implantation of the LC electrode), or treatment groups, and subsequent statistical analysis confirmed that these cohorts did not differ from one another in terms of their learning performance with regard to working, reference memory, velocity and trial latencies.
