# Supplementary material for: The Modulation by the Locus Coeruleus of Recent and Remote Memory Retrieval is Activity‐Dependent
Source: Hippocampus. 2025 Feb 20;35(2):e70004. doi: 10.1002/hipo.70004 (PMC11842585; doi:10.1002/hipo.70004)
Supplement: Supplementary file 2 — Figure S2. Histological verification and reconstruction of the electrode placement in LC. [file HIPO-35-0-s001.pdf]

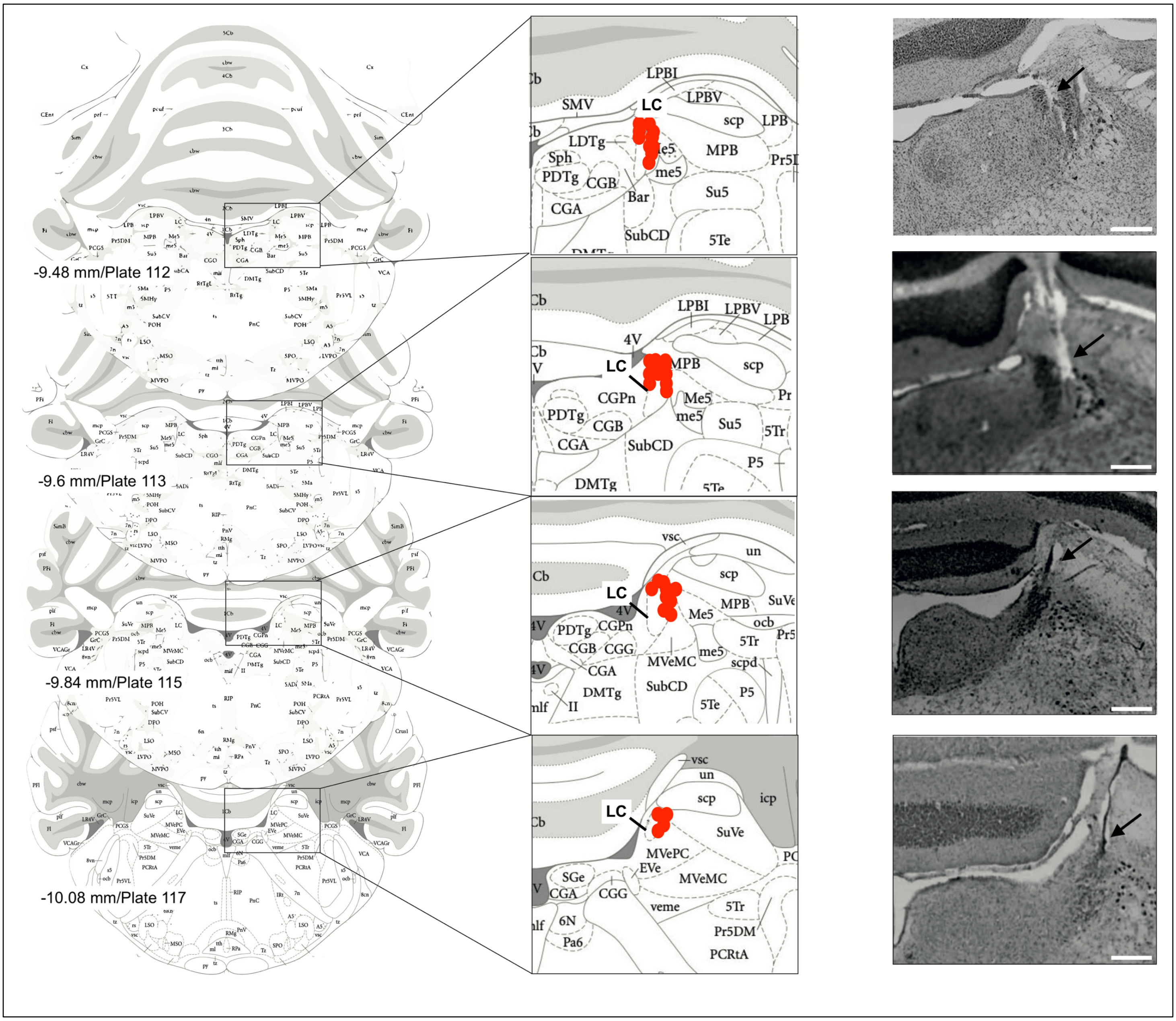

Supplementary Figure 2. Histological verification and reconstruction of the electrode placement in LC.

Left panel: Coronal sections of the rat brain (adapted from Paxinos and Watson, 2005) in which boxes outline the LC. Coordinates (in mm) anterior from Bregma, as well as the corresponding plate number in the Paxinos rat brain atlas, are shown for each example.

Middle panel: Zoom out of boxes outlining the LC, showing reconstruction of the electrode placement. The red dots represent the electrode tip location for each animal.

Right panel: Histological examples of electrode localization in LC. Brain sections show a Nissl-stained histological section of the LC. The black arrow indicates the electrode tip within the LC. Horizontal scale bar corresponds to 200 μm.
